# Esophageal Dysmotility in Multiple System Atrophy: A Retrospective Cross-Sectional Study

**DOI:** 10.3390/jcm13175026

**Published:** 2024-08-25

**Authors:** Rumi Ueha, Misaki Koyama, Akiko Seto, Taku Sato, Takao Goto, Kenta Orimo, Jun Mitsui, Tatsuya Yamasoba

**Affiliations:** 1 Swallowing Center, The University of Tokyo Hospital, Tokyo 113-8655, Japan; 2 Department of Otolaryngology and Head and Neck Surgery, Faculty of Medicine, The University of Tokyo, Tokyo 113-8655, Japan; m.sekiguchi.tymc9@gmail.com (M.K.); taku.koro.z@gmail.com (T.S.); gottytakao@gmail.com (T.G.); tyamasoba-tky@umin.ac.jp (T.Y.); 3 Department of Neurology, Faculty of Medicine, The University of Tokyo, Tokyo 113-8655, Japan; seto-ngs@umin.ac.jp (A.S.); gokudogunma@gmail.com (K.O.); mituij-tky@g.ecc.u-tokyo.ac.jp (J.M.)

**Keywords:** multiple system atrophy, esophageal motility disorders, upper esophageal sphincter, high-resolution manometry, Chicago Classification

## Abstract

**Background/Objective:** Multiple system atrophy (MSA) is often associated with dysphagia and esophageal dysmotility (ED). However, ED in patients with MSA is poorly understood. To assess the relationship between ED, dysphagia, and other clinical findings in such patients and investigate the details of ED in MSA using high-resolution manometry (HRM). **Methods:** Patients from The University of Tokyo Hospital with MSA who underwent swallowing examinations, esophagography, and HRM between 2017 and 2022 were enrolled. A retrospective chart review of patients’ backgrounds, swallowing function, and esophageal motility was performed. ED was evaluated using the Chicago Classification version 4.0. **Results:** Seventy-four patients with MSA were identified. The median age was 64 years, 48 patients (65%) were male, and the cerebellar variant type was predominant (69%). Abnormal upper esophageal sphincter (UES) resting pressure was observed in 34 patients (46%) and intraesophageal stasis in 65 (88%). High-severity MSA was a risk factor for developing dysphagia, vocal fold movement impairment, and abnormal UES function (*p* < 0.05). However, no overt clinical risk factors for ED were identified. Various types of ED were detected using HRM, and ineffective esophageal motility was the most frequent disorder. **Conclusions:** ED is a common occurrence in patients with MSA. Although a high-severity MSA may be a risk factor for developing dysphagia and vocal fold motion impairment, ED can occur regardless of clinical severity. Since ED is rarely detected based on subjective symptoms, careful evaluation of esophageal motility by esophagography or HRM is warranted in patients with MSA.

## 1. Introduction

Multiple system atrophy (MSA) is a rare and progressive neurodegenerative disorder characterized by its impact on various areas of the nervous system, encompassing both the brain and autonomic nervous systems. MSA is classified into parkinsonian (MSA-P) and cerebellar (MSA-C) variants [1]. Progressive degeneration of nerve cells and loss of nerve fibers can lead to various symptoms, including problems with movement and coordination, bladder control, and sexual function; tremors; speech difficulties; and issues with swallowing and breathing [2,3]. There is no established treatment for MSA; treatment is primarily supportive care aimed at managing symptoms and improving patients’ quality of life [3,4]. Respiratory infections and sudden death during sleep are well-known causes of death in MSA [5,6], and studies have speculated that food and fluid regurgitation during sleep may induce respiratory compromise [7,8,9].

Esophageal motility is governed by the vagus nerve, and the vagal afferent pathway is essential for the central regulation of peristalsis [10,11]. Considering the involvement of the autonomic system in MSA [12], patients with MSA may experience esophageal dysmotility (ED) because of changes in pressure and motility within the esophagus. Only few studies have reported esophageal involvement in patients with MSA [8,9]. Our group has previously reported a high incidence of ED, abnormal upper esophageal sphincter (UES) resting pressure, and abnormal deglutitive proximal esophageal contraction (ADPEC) in pharyngeal high-resolution manometry (pHRM) [9,13], and a high prevalence of intraesophageal stasis (IES) and intraesophageal reflux (IER) in videofluorographic esophagrams (VFEs) in patients with MSA [9]. However, the relationship between ED, dysphagia, and other clinical findings in patients with MSA remains poorly understood.

VFEs are a commonly used method to evaluate esophageal motility. However, detailed dynamic assessment of esophageal motility disorders using HRM has recently become increasingly common [14]. Both these tests are recommended when assessing esophageal motility because the pathologies evaluated using VFEs and HRM differ. The Chicago Classification is commonly used in the gastroenterology field [15] for esophageal motility assessment using esophageal HRM (eHRM), and it is currently in its fourth edition (2020) [16]. This classification provides a standardized approach for the analysis and categorization of ED and divides ED into two types: “disorders of the esophagogastric junction outflow” and “disorders of peristalsis”. Disorders of the esophagogastric junction outflow can be further classified into four types: types 1–3 achalasia and esophagogastric junction outflow obstruction. Peristaltic disorders include absent contractility, distal esophageal spasms, hypercontractile esophagus, and ineffective esophageal motility (IEM) [16]. There have been several reports regarding ED in patients with MSA in VFEs [8,9], but none have used eHRM.

This study aimed to assess how prevalent ED is in patients with MSA and investigate the impact of clinical factors, including disease severity, vocal fold motion impairment (VFMI), and swallowing dysfunction. Then, we examined details of ED in MSA using eHRM.

## 2. Materials and Methods

### 2.1. Patients and Ethics

**Figure 1:**
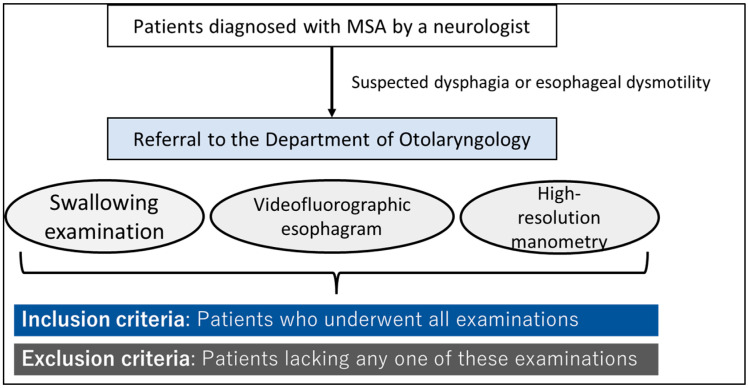
Inclusion and exclusion criteria.

Only patients diagnosed with MSA at the Department of Neurology and referred to the Department of Otolaryngology at The University of Tokyo Hospital for a swallowing evaluation between 2017 and 2022 were included, as were those who underwent all swallowing examinations, VFEs, and HRM (pharyngeal or esophageal) (Figure 1). Patient diagnoses were confirmed according to the consensus statement of the diagnostic criteria and excluded based on Gilman’s criteria [1]. The Human Ethics Committee of The University of Tokyo (2487 and 2022179NI) approved this study. All patients provided written informed consent, and their anonymity was preserved. This study was conducted following the ethical guidelines established by the 1964 Declaration of Helsinki and its subsequent amendments.

### 2.2. Methodology

This study was a retrospective, single-center analysis based on medical charts from a hospital database. First, the analysis focused on clinical and demographic details, such as age, sex, and type of disease (MSA-C or MSA-P), disease duration, the severity level of MSA [17], use of prokinetic agents, the presence of VFMI, the presence of tracheostoma, the penetration–aspiration scale (PAS) score [18] on thin liquid (10 mPa·s, 5 mL), the functional oral intake scale (FOIS) [19], HRM parameters, and VFE findings. At our institution, VFEs and HRM are performed within one week of the swallowing endoscopic evaluation. Clinical findings were evaluated from medical charts at time points within 1 month before and after these examinations. The severity of MSA was assessed on a scale of 1 to 5 using the unified multiple system atrophy rating scale (UMSARS) part IV [20] (Table 1). Resting UES, impaired UES opening during swallowing, and ADPEC were examined as HRM parameters [9,13]. VFE findings such as IES, IER, and gastroesophageal reflux (GER) were evaluated [8,21]. Using the PAS [18] and FOIS [19], scores of 1–8 (most severe score: 8) and levels of 1–7 (normal level: 7) were assigned, respectively (Table 1).

Second, partial correlations between the clinical profiles (age, sex, MSA type, disease severity, PAS scores, VFMI, IES, resting UES, impaired UES opening, and ADPEC) were statistically evaluated. Then, we statistically validated involvement of clinical factors in each of the abnormal esophageal findings, such as abnormal resting UES pressure, impaired UES opening during the swallow, ADPEC, and severe IES (grade ≥ 2) in HRM.

Finally, the type and frequency of esophageal motility disorders, based on the Chicago Classification version 4.0 [16], were investigated in patients whose esophageal motility was evaluated using eHRM.

### 2.3. HRM Study

**Figure 2:**
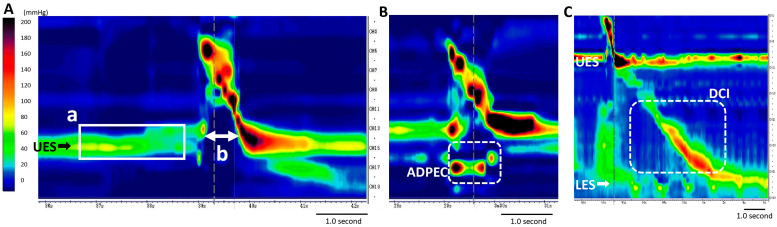
(**A**) Pharyngeal high-resolution manometry. a: resting upper esophageal sphincter (UES) pressure. b: UES opening during swallowing. (**B**) Abnormal hypertensive and discoordinated proximal esophageal contraction during swallowing (ADPEC). (**C**) Esophageal high-resolution manometry. DCI, distal contractile integral; LES, lower esophageal sphincter.

As previously reported [9,13], HRM studies were performed on all patients in the upright position. An HRM catheter (Unisensor, Portsmouth, NH, USA) was lubricated with 4% viscous lidocaine, inserted transnasally, and positioned from the oropharynx to the esophagus (Starlet; Star Medical, Tokyo, Japan). The manometric catheter had an outer diameter of 4 mm, and 20 (for pHRM) or 36 (for eHRM) circumferential pressure sensors were spaced 1 cm apart. A videofluoroscopic swallowing study (VFSS) was simultaneously carried out in the lateral view. The study protocol included a 5 min baseline recording, followed by 3 dry swallows and 3 wet swallows for pHRM or 3 dry swallows and 10 wet swallows for eHRM using 5 mL of contrast agent (iohexol: Omnipaque^®^, Daiichi-Sankyo, Tokyo, Japan). pHRM was implemented from 2017 to 2019, and eHRM was implemented starting in 2020 at our facility. Although the catheter for pHRM was too short to evaluate the entire esophagus, HRM values of proximal esophageal measures, such as resting UES pressure, UES opening duration during swallowing, and the proximal esophageal contraction integral, were obtained for all patients using either pHRM or eHRM (Figure 2A).

Abnormal HRM metrics in the UES and proximal esophagus were identified using the following criteria: abnormal resting UES pressure, hypertensive resting UES pressure (>104 mmHg), hypotensive resting UES pressure (<34 mmHg), and impaired UES relaxation pressure/duration (relaxation pressure > 12 mmHg/< 0.45 s) [13,22,23]. ADPEC was previously defined as abnormal hypertensive and discoordinated proximal esophageal contractions during swallowing as assessed by HRM [13]. In addition, a proximal esophageal contraction integral above 0 mmHg during UES opening was defined as abnormal (ADPEC) (Figure 2B). eHRM can evaluate lumen pressure from the hypopharynx to the lower esophagus (Figure 2C), and esophageal measures by eHRM were used to classify esophageal motility disorders according to the Chicago Classification version 4.0.

### 2.4. Videofluorographic Study

Videofluorographic studies were performed in the lateral and anteroposterior views. First, 3 swallows of 5 mL of thin liquid (10 mPa·s) were administered in the lateral view, and then the PAS score was evaluated. Next, a VFE was performed in the anteroposterior view with 3 swallows of 5 mL liquid. Swallowing was induced after the previous intraesophageal stasis was cleared during the second and third examinations. The presence and location of IES, IER, and GER were assessed.

**Figure 3:**
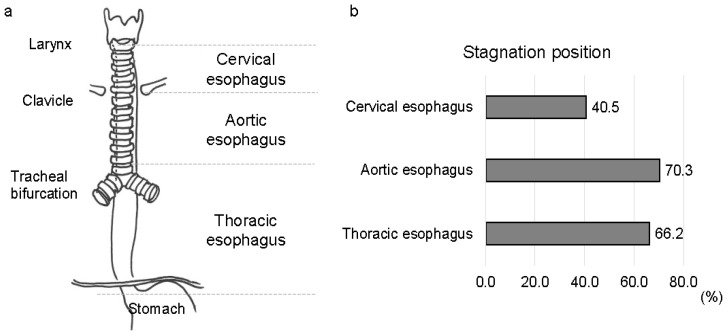
Esophageal anatomical classification and stagnation position. (**a**) Esophageal anatomical classification. (**b**) Contrast agent stagnation position in patients with multiple system atrophy.

The esophagus was anatomically divided into three sections based on fluoroscopic imaging according to previous reports [8,9,21]: (1) the cervical esophagus, proximal to the clavicles; (2) the aortic esophagus, from the clavicles distal to the aortic arch; and (3) the thoracic esophagus, from the bottom of the aortic arch to the lower esophageal sphincter (Figure 3a). IES was determined when the contrast agent failed to pass through any portion of the esophagus after the initial swallow, and after the completion of the primary peristaltic wave. The amount of contrast agent stagnation was quantified on a scale of 0 to 2: grade 0, absence of IES; grade 1, coating of contrast agent or minimal amount of contrast agent stagnation; and grade 2, stasis with a retained contrast agent that filled the lumen of the esophagus [21]. IER was present if any portion of the contrast agent traveled to a more proximal anatomical division of the esophagus [9,21]. GER is defined as any bolus that passes through the gastroesophageal junction and returns to the esophagus [9,21]. Two independent investigators performed all measurements. All patients were classified into two groups based on normal or abnormal resting UES pressure, normal or impaired UES opening during swallowing, the absence or presence of ADPEC, and IES grade (grades 0–1 or 2). Differences in the clinical features between the two groups were evaluated.

### 2.5. Statistical Analyses

Data analysis was performed using Bell Curve for Excel (version 4.04, Social Survey Research Information Co., Ltd., Tokyo, Japan). For comparing characteristics between groups, the Mann–Whitney U test was applied to continuous variables, and Fisher’s exact test, along with chi-squared tests, were used for categorical variables. *p* < 0.05 was considered statistically significant. A partial correlation analysis was performed to examine the correlation between each clinical factor. Values of 0.25–0.50 were considered a fair degree of association; 0.50–0.75, moderate to fair relation; and >0.75, good to excellent relation.

## 3. Results

### 3.1. Demographic Data and Characteristics of Patients

The demographic details of the enrolled patients are shown in Table 2. We identified 74 patients with MSA who met the eligibility criteria. The median age was 64 (interquartile range [IQR], 58–70) years, and a predominance of male patients (65%) was observed. Among the patients, 51 of 74 (69%) were diagnosed with MSA-C. The remaining 23 patients (31%) were diagnosed with MSA-P. The median disease duration was 48 (IQR, 33–66) months, and the median disease severity level was 3 (IQR, 2–4). Only four patients (4.1%) were taking oral prokinetic agents. VFMI was observed in 39 patients with MSA (53%), and tracheostomy was performed in 8 patients (11%). The median PAS score for patients with MSA was 2 (IQR, 1–5), and the median FOIS score was 7 (IQR, 5–7).

### 3.2. Esophageal Involvement in Patients with MSA

In patients with MSA, abnormal resting UES pressure was observed in 34 patients (46%); lower resting UES pressure was present in 29 patients (39%), whereas higher resting UES pressure was present in 5 patients (7%). Impaired UES opening during swallowing was found in 8 patients (11%), and ADPEC was detected in 26 patients (35%). IES was observed in 65 patients (88%): grade 0 in 9 patients (12%), grade 1 in 48 patients (65%), and grade 2 in 17 patients (23%). Regarding the location of contrast stasis, IES frequently occurred in the aortic esophagus (52 patients, 70%) and thoracic esophagus (49 patients, 66%) (Figure 3b). IER and GER were observed in 46 (62%) and 6 (8%) patients, respectively (Table 3).

### 3.3. Clinical Risk Factors for ED in Patients with MSA

Patients were classified into two groups according to the presence or absence and degree of abnormal findings related to ED to examine the clinical risk factors for ED in patients with MSA: abnormal resting UES pressure, impaired UES opening during swallowing, ADPEC, and IES grade (grades 0–1, or 2). The clinical factors listed in Table 4 were evaluated. MSA severity and penetration–aspiration were risk factors for abnormal UES function, including abnormal resting UES pressure and impaired UES opening during swallowing. MSA severity and VFMI were risk factors for ADPEC, whereas no overt clinical risk factors for IES were identified (Table 4).

**Figure 4:**
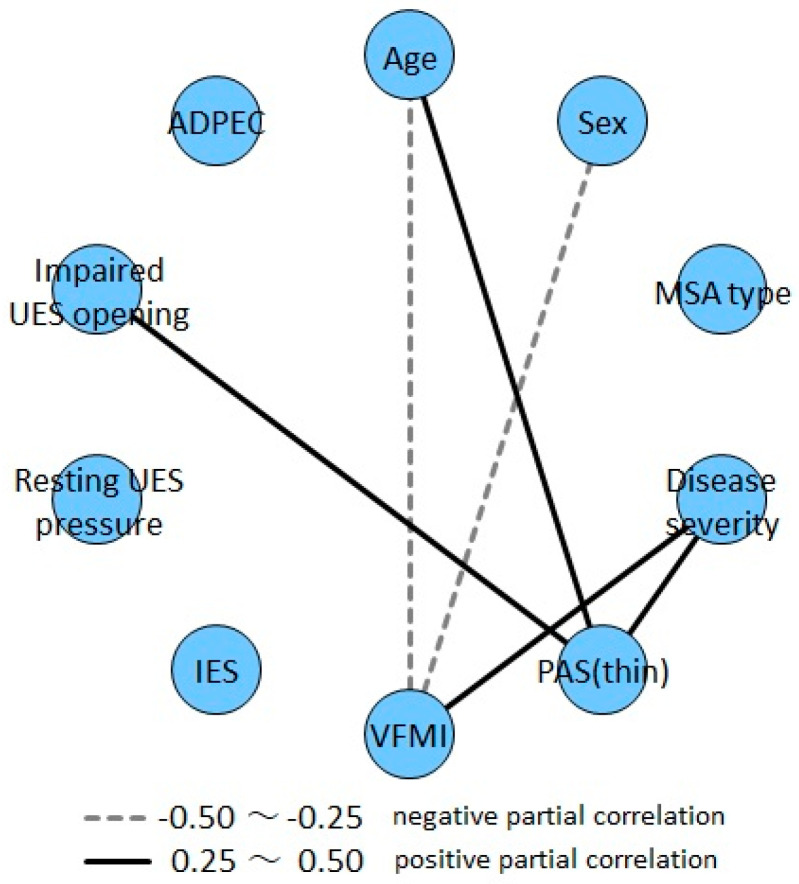
Partial correlation networks. PAS, penetration–aspiration scale; VFMI, vocal fold motion impairment; IES, intraesophageal stasis; UES, upper esophageal sphincter; ADPEC, abnormal deglutitive proximal esophageal contraction. −0.5 to −0.25: negative partial correlation, 0.25–0.5: positive partial correlation.

In addition, we investigated the correlation of each of the two factors among age, sex, MSA type, UMSARS part IV score, PAS score, VFMI, and esophageal movement disorder (abnormal resting UES pressure, UES opening disorder, and ADPEC) in patients with MSA. Partial correlation analysis was performed to determine the correlation between two variables after removing the effects of other variables. Positive correlations were observed between age and PAS, severity and VFMI, and severity and PAS, as well as between impaired UES opening during swallowing and PAS. However, no other clinical factors correlating with esophageal motility disorders were identified (Figure 4, Supplemental Table S1).

### 3.4. Various Esophageal Motility Disorders in Patients with MSA

**Figure 5:**
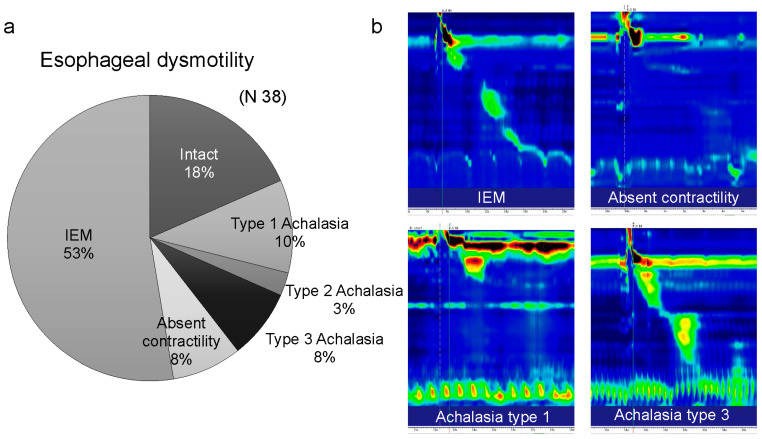
Various esophageal motility disorders in multiple system atrophy. (**a**) Proportion of various esophageal motility disorders. (**b**) Representative high-resolution manometric findings of various esophageal motility disorders. IEM, ineffective esophageal motility.

Of the 74 patients with MSA, 38 were evaluated for esophageal motility using eHRM. eHRM revealed esophageal motility disorders in 31 patients (81.6%). According to the Chicago Classification version 4.0, 20 patients had IEM, 8 had achalasia (4 Type 1, 1 Type 2, and 3 Type 3), and 3 showed absent contractility. IEM was the most frequent disorder, and achalasia, which indicates impaired lower esophageal sphincter relaxation, was found in approximately 20% of the patients (Figure 5).

## 4. Discussion

MSA is a rare, progressive condition characterized by neurodegeneration that influences the autonomic nervous system and motor functions. Esophageal involvement in MSA has rarely been examined, although swallowing dysfunction, VFMI, and sleep apnea are frequent symptoms of MSA [24]. In this study, we identified a high prevalence of ED in patients with MSA. No clinical factors, including MSA severity, were correlated with ED, except for UES function, whereas MSA severity and aspiration were risk factors for abnormal UES function. In addition, we demonstrated that patients with MSA showed various types of esophageal motility disorders according to the Chicago Classification version 4.0.

The esophagus, a tube composed of fibrous and muscular tissue, facilitates the transport of food from the hypopharynx to the stomach via peristaltic contractions. The autonomic nervous system controls esophageal peristalsis and sphincter function with contributions from the parasympathetic and sympathetic divisions through the vagus nerve. Proximal regions, including the UES, are composed of striated muscle. Peristalsis in the striated muscle is controlled by somatic nerves with cell bodies located in the brainstem, specifically in a region of the rostral nucleus ambiguus. The sequential contractions of peristalsis are orchestrated by a pattern generator circuit in the brainstem, which incorporates the nucleus of the solitary tract and is modulated by vagal afferents. The distal esophageal regions, including the lower sphincter, are composed of smooth muscle, and the central and peripheral neural systems with intramural enteric circuitry control peristalsis. The myenteric plexus of the esophagus contains the peripheral neuronal elements that mediate the esophageal off-response, lower esophageal sphincter’s relaxation, and hyperpolarization of the circular esophageal muscle cells [10,11,25]. Autonomic nervous system dysfunction leads to a decrease in the amplitude and frequency of esophageal contractions, resulting in delayed food transport from the pharynx to the stomach. This delay can cause food stagnation in the esophagus, leading to regurgitation and aspiration into the lungs. Since aging has been reported to be correlated with esophageal motor abnormalities [26], the high frequency of ED in MSA, which often occurs in middle-aged patients, should consider the influence of age.

Abnormal UES function in patients with MSA has been reported in several papers, with one reporting decreased UES pressure [27] and another reporting abnormally high UES pressure [28]. Since Parkinson’s disease is associated with an abnormally increased UES resting pressure [29], it is not surprising to observe a higher UES resting pressure in patients with MSA-P, in which parkinsonism is predominant. In this study, both abnormally high and low UES pressures were observed in patients with MSA. However, lower UES pressures were observed more frequently, which may be attributed to the higher prevalence of patients with MSA-C compared with that of MSA-P patients in this study. In addition, similar to a previous report that patients with VFMI are more likely to have UES opening [30], this study showed that VFMI is a risk factor for impaired UES opening. This result is supported by the fact that the vagal nucleus governs both vocal fold movement and UES function.

Given that MSA leads to progressive neuronal degeneration in multiple brain regions, we speculated that patients with this condition could show various pathological esophageal motility issues. To the best of our knowledge, no previous study has evaluated esophageal motility using eHRM in patients with MSA, and this is the first report describing these findings. In this study, eHRM revealed that IEM was the most frequently observed, followed by types 1–3 achalasia, which indicates inadequate relaxation of the LES and no esophageal peristalsis, suggesting that MSA can cause various esophageal motility disorders. Furthermore, hypothesizing that clinical factors might contribute to esophageal motility disorders, we examined the partial correlation between ED and clinical factors, including age, sex, MSA type (P or C), and MSA severity. However, no significant associations were observed among them. Therefore, this means that patients with MSA, even those with mild MSA severity, should be aware of ED.

Patients with ED should be cautioned against nocturnal involuntary gastroesophageal regurgitation because sudden death is the most serious consequence of MSA [31] and has been reported to be the second most common cause of death in MSA [5]. The causes of sudden death include upper airway obstruction, central respiratory disturbance, or both [32], and the causes of upper airway obstruction include floppy epiglottis, VFMI, increased sputum because of dysphagia, and aspiration and suffocation secondary to food regurgitation during sleep [24,33,34]. A report [35] of a patient with MSA who had repeated aspiration pneumonia despite complete gastrostomy nutrition and no longer developed aspiration pneumonia after endoscopic jejunostomy feeding suggests caution against pneumonia because of aspiration of subclinical gastroesophageal regurgitation in patients with MSA. Generally, in MSA, ED can occur regardless of clinical severity. Thus, medical professionals must pay close attention to ED in these patients. The appropriate evaluation of esophageal motility may prevent or delay severe complications in patients with MSA because patients with ED do not necessarily report subjective symptoms. Suppose VFEs and HRM identify ED in patients with MSA. In that case, management options may involve treatments and recommendations such as medications to facilitate gastrointestinal motility, avoiding foods that are difficult to digest, and keeping an upright or semi-upright position for a few hours after meals to reduce the likelihood of food regurgitation. Looking ahead, it would be ideal to develop an artificial intelligence-driven diagnosis [36] of ED by deep learning from questionnaire scores (e.g., the FSSG questionnaire [37]) and other key clinical factors.

This study had several limitations. First, the study’s retrospective nature is subject to selection bias, and longitudinal observations are required to elucidate the clinical significance of esophageal evaluation in patients with MSA. Second, an esophageal mucosal evaluation was not performed in most patients, although esophagitis may have influenced esophageal motility. Third, the amount of contrast agent used for the VFEs was small (5 mL), and the number of VFE evaluations was limited to reduce the potential aspiration risk. Future research on ED in patients with MSA should include longitudinal questionnaire surveys on esophageal symptoms, as well as prospective studies of esophageal evaluation, including upper gastrointestinal endoscopy, VFEs, and HRM.

## 5. Conclusions

ED is common among patients with MSA. Although a high severity of MSA may be a risk factor for developing dysphagia and VFMI, ED can occur regardless of clinical severity. Careful evaluation of esophageal motility by VFEs or HRM is warranted in patients with MSA, as subjective symptoms and physical examination rarely detect ED.

## Figures and Tables

**Table 1:** Patients’ demographic details of severity levels of multiple system atrophy (unified multiple system atrophy rating scale, part IV) [20], penetration–aspiration scale [18], functional oral intake scale [19], and grading of intraesophageal stasis of contrast agent [21].^.^

Unified multiple system atrophy rating scale, part IV: global disability scale	
Scale 1	Completely independent. Able to do all chores with minimal difficulty or impairment. Essentially normal. Unaware of any difficulty.
Scale 2	Not completely independent. Needs help with some chores.
Scale 3	More dependent. Help with half of chores. Spends a large part of the day with chores.
Scale 4	Very dependent. Now and then does a few chores alone or begins alone. Much help needed.
Scale 5	Totally dependent and helpless. Bedridden.
Penetration–aspiration scale	
Score 1	Material does not enter the airway.
Score 2	Material enters the airway, remains above the vocal folds, and is ejected from the airway.
Score 3	Material enters the airway, remains above the vocal folds, and is not ejected from the airway.
Score 4	Material enters the airway, contacts the vocal folds, and is ejected from the airway.
Score 5	Material enters the airway, contacts the vocal folds, and is not ejected from the airway.
Score 6	Material enters the airway, passes below the vocal folds, and is ejected into the larynx or out of the airway.
Score 7	Material enters the airway, passes below the vocal folds, and is not ejected from the trachea despite effort.
Score 8	Material enters the airway, passes below the vocal folds, and no effort is made to eject.
Functional oral intake scale	
Level 1	No oral intake.
Level 2	Tube dependent with minimal/inconsistent oral intake.
Level 3	Tube supplements with consistent oral intake.
Level 4	Total oral intake of a single consistency.
Level 5	Total oral intake of multiple consistencies requiring special preparation.
Level 6	Total oral intake with no special preparation but must avoid specific foods or liquid items.
Level 7	Total oral intake with no restrictions.
Grading of intraesophageal stasis of contrast agent	
Grade 0	Absence of intraesophageal stasis.
Grade 1	A minimal amount of contrast agent stagnation or a coating of contrast agent.
Grade 2	Stagnation with contrast agent that completely filled the lumen of the esophagus.

**Table 2:** Demographic and clinical data.

Characteristic	
Patients-no.	74
Age-years, median (IQR)	64 (58–70)
Men-no. (%)	48 (64.9)
MSA-C: MSA-P (no.)	51:23
MSA disease duration-mth. median (IQR)	48 (33–66)
Disease severity level	3 (2–4)
Use of prokinetic agents-no. (%)	4 (4.1)
Vocal fold motion impairment-no. (%)	39 (52.7)
Tracheostomy-no. (%)	8 (10.8)
PAS score. median (IQR)	2 (1–5)
FOIS score. median (IQR)	7 (5–7)

no., number; IQR, interquartile range; MSA, multiple system atrophy; MSA-C, cerebellar variant type of multiple system atrophy; MSA-P, parkinsonian variant type of multiple system atrophy; PAS, penetration–aspiration scale; FOIS, functional oral intake scale.

**Table 3:** Esophageal involvement in all patients.

Esophageal Involvement		
Abnormal resting UES pressure-no. (%)		34 (45.9)
	Higher UES pressure	5 (6.8)
	Lower UES pressure	29 (39.2)
Impaired UES opening-no. (%)		8 (10.8)
ADPEC-no. (%)		26 (35.1)
Intraesophageal stasis	Grade median (IQR)	1 (1–1)
	Grade 0-no. (%)	9 (12.2)
	Grade 1-no. (%)	48 (64.9)
	Grade 2-no. (%)	17 (23.0)
Intraesophageal reflux-no. (%)		46 (62.2)
Gastroesophageal reflux-no. (%)		6 (8.1)

UES, upper esophageal sphincter; no., number; ADPEC, abnormal deglutitive proximal esophageal contraction; IQR, interquartile range.

**Table 4:** Clinical risk factors for esophageal dysmotility in patients with multiple system atrophy.

	**Resting UES Pressure**			**UES Opening during Swallow**		
	**Normal**	**Abnormal**	***p* Value**	**Normal**	**Impaired**	***p* Value**
Number (%)	40 (54.1)	34 (45.9)		66 (89.2)	8 (10.8)	
Age-years, median (IQR)	64 (55–69)	65 (60–71)	0.14	64 (58–70)	62 (55–67)	0.58
Men-no. (%)	19 (55.9)	29 (72.5)	0.14	44 (66.7)	4 (50.0)	0.35
MSA-C: MSA-P (no.)	C 20, P 14	C 31, P 9	0.08	C 47, P 19	C 4, P 4	0.22
Disease severity level	3 (2–4]	3 (3–4]	0.038 *	3 (2–4)	4 (4–5)	0.0013 **
Vocal fold motion impairment-no. (%)	20 (58.8)	19 (47.5)	0.33	33 (50.0)	6 (75.0)	0.18
PAS score. median (IQR)	1 (1–3)	3 (1–7)	0.034 *	1 (1–3)	8 (5–8)	<0.001 ***
	**ADPEC**			**IES**		
	**Absent**	**Present**	***p* Value**	**Grade 0–1**	**Grade 2**	***p* Value**
Number (%)	48 (64.9)	26 (35.1)		57 (77.0)	17 (23.0)	
Age-years, median (IQR)	66 (59–73)	63 (56–65)	0.047 *	63 (57–71)	67 (63–70)	0.28
Men-no. (%)	31 (64.6)	17 (65.4)	0.95	22 (61.4)	13 (76.5)	0.25
MSA-C: MSA-P (no.)	C 35, P 15	C 18, P 8	0.97	C 39, P 18	C 12, P 5	0.87
Disease severity level	3 (2–4)	3 (3–4)	0.0035 **	3 (2–4)	3 (3–4)	0.32
Vocal fold motion impairment-no. (%)	20 (41.7)	19 (73.1)	0.001 **	28 (49.1)	11 (64.7)	0.26
PAS score. median (IQR)	1 (1–5)	3 (1–6)	0.30	2 (1–5)	1 (1–6)	0.32

UES, upper esophageal sphincter; IQR, interquartile range; no., number; MSA-C, cerebellar variant type of multiple system atrophy; MSA-P, parkinsonian variant type of multiple system atrophy; PAS, penetration–aspiration scale; ADPEC, abnormal deglutitive proximal esophageal contraction; IES, intraesophageal stasis. *: *p* < 0.05; **: *p* < 0.01; ***: *p* < 0.001.

## Data Availability

The datasets used and analyzed during the current study are available from the corresponding author on reasonable request.

## Supplementary Materials

The following supporting information can be downloaded at: https://www.mdpi.com/article/10.3390/jcm13175026/s1, Table S1. Partial correlation coefficient.

## Abbreviations

MSA	multiple system atrophy
ED	esophageal dysmotility
HRM	high-resolution manometry
UES	upper esophageal sphincter
MSA-P	multiple system atrophy parkinsonian variant
MSA-C	multiple system atrophy cerebellar variant
ADPEC	abnormal deglutitive proximal esophageal contraction
pHRM	pharyngeal high-resolution manometry
VFE	videofluorographic esophagram
IES	intraesophageal stasis
IER	intraesophageal reflux
eHRM	esophageal high-resolution manometry
VFMI	vocal fold motion impairment
PAS	penetration–aspiration scale
FOIS	functional oral intake scale
UMSARS	unified multiple system atrophy rating scale
GER	gastroesophageal reflux
VFSS	videofluoroscopic swallowing study
IQR	interquartile range

## References

[1] Gilman S., Wenning G., Low P.A., Brooks D., Mathias C.J., Trojanowski J.Q., Wood N., Colosimo C., Durr A., Fowler C.J. (2008). Second consensus statement on the diagnosis of multiple system atrophy. Neurology.

[2] Fanciulli A., Wenning G.K. (2015). Multiple-system atrophy. N. Engl. J. Med..

[3] Poewe W., Stankovic I., Halliday G., Meissner W.G., Wenning G.K., Pellecchia M.T., Seppi K., Palma A., Kaufmann H. (2022). Multiple system atrophy. Nat. Rev. Dis. Primers.

[4] Flabeau O., Meissner W.G., Tison F. (2010). Multiple system atrophy: Current and future approaches to management. Ther. Adv. Neurol. Disord..

[5] Zhang L., Cao B., Zou Y., Wei Q.-Q., Ou R., Liu W., Zhao B., Yang J., Wu Y., Shang H. (2018). Causes of death in Chinese patients with multiple system atrophy. Aging Dis..

[6] Papapetropoulos S., Tuchman A., Laufer D., Papatsoris A.G., Papapetropoulos N., Mash D.C. (2007). Causes of death in multiple system atrophy. J. Neurol. Neurosurg. Psychiatry.

[7] Davis P.K., Remick D.G., Parascandola S.A., Spangler S., Wise R.K., Martin L.F. (1989). Intravascular plastic catheters potentiate tumor necrosis factor release and cardiac dysfunction secondary to infection. Curr. Surg..

[8] Taniguchi H., Nakayama H., Hori K., Nishizawa M., Inoue M., Shimohata T. (2015). Esophageal involvement in multiple system atrophy. Dysphagia.

[9] Ueha R., Sato T., Goto T., Yamauchi A., Nativ-Zeltzer N., Mitsui J., Belafsky P.C., Yamasoba T. (2021). Esophageal dysmotility is common in patients with multiple system atrophy. Laryngoscope.

[10] Park H., Conklin J.L. (1999). Neuromuscular control of esophageal peristalsis. Curr. Gastroenterol. Rep..

[11] Chen J.H. (2016). Ineffective esophageal motility and the vagus: Current challenges and future prospects. Clin. Exp. Gastroenterol..

[12] Coon E.A., Cutsforth-Gregory J.K., Benarroch E.E. (2018). Neuropathology of autonomic dysfunction in synucleinopathies. Mov. Disord..

[13] Ueha R., Goto T., Sato T., Nativ-Zeltzer N., Shen S.C., Nito T., Belafsky P.C., Yamasoba T. (2018). High resolution manofluorographic study in patients with multiple system atrophy: Possible early detection of upper esophageal sphincter and proximal esophageal abnormality. Front. Med..

[14] Aspirot A., Faure C. (2013). Esophageal dysmotility: Characterization and pathophysiology. Dis. Esophagus.

[15] Bredenoord A.J., Fox M., Kahrilas P.J., Pandolfino J.E., Schwizer W., Smout A.J.P.M., International High Resolution Manometry Working Group (2012). Chicago classification criteria of esophageal motility disorders defined in high resolution esophageal pressure topography. Neurogastroenterol. Motil.

[16] Yadlapati R., Kahrilas P.J., Fox M.R., Bredenoord A.J., Prakash Gyawali C., Roman S., Babaei A., Mittal R.K., Rommel N., Savarino E. (2021). Esophageal motility disorders on high-resolution manometry: Chicago classification version 4.0©. Neurogastroenterol. Motil..

[17] Watanabe H., Saito Y., Terao S., Ando T., Kachi T., Mukai E., Aiba I., Abe Y., Tamakoshi A., Doyu M. (2002). Progression and prognosis in multiple system atrophy: An analysis of 230 Japanese patients. Brain.

[18] Rosenbek J.C., Robbins J.A., Roecker E.B., Coyle J.L., Wood J.L. (1996). A penetration-aspiration scale. Dysphagia.

[19] Crary M.A., Mann G.D.C., Groher M.E. (2005). Initial psychometric assessment of a functional oral intake scale for dysphagia in stroke patients. Arch. Phys. Med. Rehabilit..

[20] Wenning G.K., Tison F., Seppi K., Sampaio C., Diem A., Yekhlef F., Ghorayeb I., Ory F., Galitzky M., Scaravilli T. (2004). Development and validation of the Unified Multiple System Atrophy Rating Scale (UMSARS). Mov. Disord..

[21] Jou J., Radowsky J., Gangnon R., Sadowski E., Kays S., Hind J., Gaumnitz E., Taylor A., Robbins J. (2009). Esophageal clearance patterns in normal older adults as documented with videofluoroscopic esophagram. Gastroenterol. Res. Pract..

[22] Mathews S.C., Ciarleglio M., Chavez Y.H., Clarke J.O., Stein E., Chander Roland B. (2014). Upper esophageal sphincter abnormalities are strongly predictive of treatment response in patients with achalasia. World J. Clin. Cases.

[23] Park C.-H., Lee Y.-T., Yi Y., Lee J.-S., Park J.H., Yoon K.J. (2017). Ability of high-resolution manometry to determine feeding method and to predict aspiration pneumonia in patients with dysphagia. Am. J. Gastroenterol..

[24] Tsuchiya K., Ueha R., Suzuki S., Goto T., Sato T., Nito T., Yamasoba T. (2020). Heightened risk of early vocal fold motion impairment onset and dysphagia in the parkinsonian variant of multiple system atrophy: A comparative study. Clin. Park. Relat. Disord..

[25] Richards W.G., Sugarbaker D.J. (1995). Neuronal control of esophageal function. Chest Surg. Clin. N. Am..

[26] Gutschow C.A., Leers J.M., Schröder W., Prenzel K.L., Fuchs H., Bollschweiler E., Bludau M., Hölscher A.H. (2011). Effect of aging on esophageal motility in patients with and without GERD. Ger. Med. Sci..

[27] Park H.W., Jung H.Y., Lee D., Kim D.H., Jung K.W., Chung J.W., Choi K.S., Choi K.D., Song H.J., Kim J.H. (2008). A case of multiple system atrophy presenting with esophageal dysphagia. Korean J. Neurogastroenterol. Motil..

[28] Alfonsi E., Versino M., Merlo I.M., Pacchetti C., Martignoni E., Bertino G., Moglia A., Tassorelli C., Nappi G. (2007). Electrophysiologic patterns of oral-pharyngeal swallowing in parkinsonian syndromes. Neurology.

[29] Higo R., Tayama N., Watanabe T., Niimi S. (2001). Abnormal elevation of resting pressure at the upper esophageal sphincter of Parkinson’s disease patients. Eur. Arch. Otorhinolaryngol..

[30] Higo R., Tayama N., Watanabe T., Nitou T., Takeuchi S. (2003). Vocal fold motion impairment in patients with multiple system atrophy: Evaluation of its relationship with swallowing function. J. Neurol. Neurosurg. Psychiatry.

[31] Shimohata T., Aizawa N., Nakayama H., Taniguchi H., Ohshima Y., Okumura H., Takahashi T., Yokoseki A., Inoue M., Nishizawa M. (2016). Mechanisms and prevention of sudden death in multiple system atrophy. Park. Relat. Disord..

[32] Ozawa T., Sekiya K., Aizawa N., Terajima K., Nishizawa M. (2016). Laryngeal stridor in multiple system atrophy: Clinicopathological features and causal hypotheses. J. Neurol. Sci..

[33] Isozaki E., Naito A., Horiguchi S., Kawamura R., Hayashida T., Tanabe H. (1996). Early diagnosis and stage classification of vocal cord abductor paralysis in patients with multiple system atrophy. J. Neurol. Neurosurg. Psychiatry.

[34] Shepherd K., Hillman D., Eastwood P. (2013). Symptoms of aerophagia are common in patients on continuous positive airway pressure therapy and are related to the presence of nighttime gastroesophageal reflux. J. Clin. Sleep Med..

[35] Ogawa T., Oodaira H., Suzuki Y., Hashimoto R., Kato H. (2009). Successive application of percutaneous endoscopic gastrostomy with jejunal extension and percutaneous endoscopic jejunostomy in a case of multiple system atrophy. Rinsho Shinkeigaku.

[36] Taciuc I.-A., Dumitru M., Vrinceanu D., Gherghe M., Manole F., Marinescu A., Serboiu C., Neagos A., Costache A. (2024). Applications and challenges of neural networks in otolaryngology (Review). Biomed. Rep..

[37] Kusano M., Shimoyama Y., Sugimoto S., Kawamura O., Maeda M., Minashi K., Kuribayashi S., Higuchi T., Zai H., Ino K. (2004). Development and evaluation of FSSG: Frequency scale for the symptoms of GERD. J. Gastroenterol..

