# Supplementary material for: Esophageal Dysmotility in Multiple System Atrophy: A Retrospective Cross-Sectional Study
Source: J Clin Med. 2024 Aug 25;13(17):5026. doi: 10.3390/jcm13175026 (PMC11396424; doi:10.3390/jcm13175026)
Supplement: Supplementary file 1 [file jcm-13-05026-s001.zip › jcm-3153444-supplementary.pdf]

Supplemental Table S1. Partial correlation coefficient.

	Age	Sex	MSA type	Disease severity	PAS (thin)	VFMI	IES	Abnormal resting UES pressure	Impaired UES opening	ADPEC
Age	1.0000	0.0388	0.1038	0.0737	0.3379	-0.2800	-0.0923	0.0903	-0.1643	-0.1395
Sex		1.0000	-0.0751	0.0111	0.0503	-0.2751	-0.0266	0.2020	0.1005	0.0837
MSA type			1.0000	-0.0853	-0.0469	-0.0021	0.0530	0.2132	0.1832	0.0120
Disease severity				1.0000	0.2689	0.4099	0.1321	0.1657	0.2225	0.0720
PAS (thin)					1.0000	0.0595	0.0207	0.0991	0.3336	-0.0082
VFMI						1.0000	0.0806	0.1213	-0.0704	0.1818
IES							1.0000	-0.0217	-0.0862	0.0315

Abnormal resting UES pressure								1.0000	-0.1087	-0.1491
Impaired UES opening									1.0000	0.1979
ADPEC										1.0000

UES, upper esophageal sphincter; MSA, multiple system atrophy; PAS, Penetration Aspiration Scale; ADPEC, abnormal deglutitive proximal esophageal contraction; IES, intraesophageal stasis; VFMI, vocal-fold motion impairment
